# Além da Remissão: O Impacto Cardiometabólico da Vasculite Associada a ANCA

**DOI:** 10.36660/abc.20260373

**Published:** 2026-06-16

**Authors:** Manuela Fiuza

**Affiliations:** 1 Serviço de Cardiologia ULSSM, CAML, CCUL@RISE, FMUL Lisboa Portugal Serviço de Cardiologia ULSSM, CAML, CCUL@RISE, FMUL, Lisboa - Portugal

**Keywords:** Síndrome Metabólica, Vasculite, Doenças Reumáticas, Índice de Massa Corporal

A intersecção entre inflamação sistêmica e doença cardiometabólica emergiu como um paradigma definidor na medicina contemporânea. As vasculites associadas a anticorpos anticitoplasma de neutrófilos (ANCA) (VAA) — incluindo granulomatose com poliangeíte (GPA), poliangeíte microscópica (PAM) e granulomatose eosinofílica com poliangeíte (GEPA) — representam um modelo particularmente convincente, no qual a lesão vascular imunomediada coexiste com uma carga metabólica substancial e frequentemente subdiagnosticada. Nesta edição, Bezerra et al.^1^ fornecem dados importantes sobre a prevalência e os preditores da síndrome metabólica (SM) nessa população.

Utilizando os critérios do NCEP ATP III, os autores relatam uma prevalência de SM de 43,5% em uma coorte de 62 pacientes consecutivos com VAA acompanhados em um centro terciário de referência brasileiro, em comparação com apenas 13,2% em controles pareados por idade e sexo (p=0,001). Na análise multivariada restrita a pacientes com VAA, o sexo masculino (OR=11,43; IC 95%=2,81–46,52) e o índice de massa corporal mais elevado (OR=1,13; IC 95%=1,02–1,25) emergiram como preditores independentes de SM. Esses achados estão em consonância com relatos anteriores em populações com VAA, onde a prevalência de SM variou de 43% a 51%,^2-4^ refletindo padrões observados em um amplo espectro de doenças reumáticas autoimunes sistêmicas, incluindo lúpus eritematoso sistêmico, artrite reumatoide e esclerose sistêmica.^5,6^

Uma contribuição crucial deste estudo é a dissociação fenotípica observada: embora o IMC não tenha diferido entre pacientes e controles, os pacientes com VAA apresentaram circunferência da cintura significativamente maior, indicando uma predominância de adiposidade central. A gordura visceral tem implicações cardiometabólicas mais fortes do que a obesidade generalizada — estando mais intimamente ligada à resistência à insulina, inflamação sistêmica e aterosclerose acelerada — e pode ser impulsionada, pelo menos em parte, pela redistribuição de gordura induzida por glicocorticoides. Essa descoberta questiona o uso exclusivo do IMC como marcador de risco nessa população e apoia a incorporação rotineira da medição da circunferência da cintura na avaliação clínica.

A forte associação da SM com o sexo masculino (OR >11) é uma descoberta intrigante e clinicamente importante. Paradoxalmente, os pacientes com SM nesta coorte eram mais jovens do que aqueles sem a síndrome, uma observação que pode refletir um viés de encaminhamento para pacientes mais gravemente afetados em centros terciários, exposição imunossupressora mais precoce e intensiva em indivíduos mais jovens e a crescente prevalência de SM entre homens mais jovens, documentada em dados epidemiológicos contemporâneos.^7^ Em conjunto, esses achados defendem a vigilância cardiometabólica sistemática em todas as faixas etárias na VAA, em vez de reservar o rastreamento para pacientes mais velhos.

A relação entre VAA e SM é multifatorial. A inflamação sistêmica crônica promove disfunção endotelial, estresse oxidativo e dislipidemia. Paralelamente, a terapia com glicocorticoides — ainda um pilar do tratamento da VAA — contribui para o ganho de peso, resistência à insulina, hipertensão e perfis lipídicos adversos. Embora o estudo não tenha identificado o uso atual de prednisona como um preditor independente de SM, a ausência de dados de exposição cumulativa impede conclusões definitivas. Notavelmente, a maior frequência de uso de micofenolato de mofetila entre pacientes com SM foi atribuída corretamente a viés de indicação, e não a qualquer efeito diabetogênico ou pró-aterogênico direto do medicamento — uma distinção metodológica importante para futuros estudos que abordem padrões de tratamento e consequências metabólicas.

O estudo também destaca que a própria VAA — juntamente com um histórico familiar positivo para doença cardiovascular — constitui um fator independente associado à SM, reforçando a ideia de que pacientes com vasculite não apenas acumulam fatores de risco tradicionais em taxas mais elevadas, mas que o ambiente específico da doença amplifica substancialmente o perfil cardiometabólico geral. É importante ressaltar que a prevalência de SM não diferiu de acordo com a atividade da doença ou o subtipo de vasculite, sugerindo que o risco cardiometabólico é relativamente independente do fenótipo da vasculite e deve ser abordado em todos os pacientes, independentemente das características da doença.

O estudo apresenta limitações importantes que os autores reconhecem de forma transparente. Seu delineamento transversal impede inferências causais, e a amostra de conveniência de um único centro limita a generalização dos resultados. O tamanho relativamente pequeno da amostra restringe o ajuste multivariável para fatores de confusão relevantes, particularmente a exposição cumulativa a glicocorticoides. Estudos prospectivos, multicêntricos e longitudinais são necessários para esclarecer a dinâmica temporal entre a atividade da VAA, o tratamento e o desenvolvimento da SM, e para avaliar se as intervenções direcionadas à SM se traduzem em redução das taxas de eventos cardiovasculares nessa população.^8-13^

As implicações clínicas são claras. A triagem sistemática dos componentes da SM — pressão arterial, glicemia de jejum, perfil lipídico e circunferência abdominal — deve se tornar prática padrão no monitoramento de pacientes com a VAA. O manejo precoce e agressivo dos fatores de risco modificáveis, incluindo intervenções no estilo de vida e estratégias para reduzir o uso de glicocorticoides quando viável, deve ser integrado à rotina de atendimento. Cardiologistas e reumatologistas devem colaborar estreitamente para reduzir a carga cardiovascular a longo prazo nesses pacientes.

Em conclusão, Bezerra et al.^1^ reforçam que a VAA não é apenas uma doença vasculítica, mas também uma doença cardiometabólica. A alta prevalência da SM — associada ao sexo masculino, obesidade central, hipertensão, dislicemia e dislipidemia — exige uma abordagem de manejo holística que vai muito além do controle da atividade da doença. Este estudo é um lembrete oportuno de que a excelência no tratamento da vasculite requer a superação da lacuna entre a remissão imunológica e a redução abrangente do risco cardiovascular.
