# Supplementary material for: Além da Remissão: O Impacto Cardiometabólico da Vasculite Associada a ANCA
Source: Arq Bras Cardiol. 2026 Jun 16;123(4):e20260373. [Article in Portuguese] doi: 10.36660/abc.20260373 (PMC13398814; doi:10.36660/abc.20260373)
Supplement: Translation

# Translation: Beyond Remission: The Cardiometabolic Burden of ANCA-Associated Vasculitis

**DOI:** 10.36660/abc.20260373i

**Authors:** Manuela Fiuza

**Affiliations:** 1 Lisbon Portugal Serviço de Cardiologia ULSSM, CAML, CCUL@RISE, FMUL, Lisbon – Portugal

**Keywords:** Metabolic Syndrome, Vasculitis, Rheumatic Diseases, Body Mass Index

The intersection between systemic inflammation and cardiometabolic disease has emerged as a defining paradigm in contemporary medicine. Antineutrophil cytoplasmic antibody (ANCA)-associated vasculitides (AAV)—including granulomatosis with polyangiitis (GPA), microscopic polyangiitis (MPA), and eosinophilic granulomatosis with polyangiitis (EGPA)—represent a particularly compelling model where immune-mediated vascular injury coexists with a substantial and often underrecognized metabolic burden. In this issue, Bezerra et al.^1^ provide important data regarding the prevalence and predictors of metabolic syndrome (MetS) within this population.

Using the NCEP ATP III criteria, the authors report a MetS prevalence of 43.5% in a cohort of 62 consecutive AAV patients followed at a Brazilian tertiary referral center, compared with only 13.2% in age- and sex-matched controls (p=0.001). In multivariable analysis restricted to AAV patients, male sex (OR=11.43; 95%CI=2.81–46.52) and higher body mass index (OR=1.13; 95%CI=1.02–1.25) emerged as independent predictors of MetS. These findings align with prior reports in AAV populations, where MetS prevalence has ranged from 43% to 51%,^2-4^ mirroring patterns seen across a broad spectrum of systemic autoimmune rheumatic diseases, including systemic lupus erythematosus, rheumatoid arthritis, and systemic sclerosis.^5,6^

A critical contribution of this study is the phenotypic dissociation observed: although BMI did not differ between patients and controls, AAV patients exhibited significantly greater waist circumference, indicating a predominance of central adiposity. Visceral fat carries stronger cardiometabolic implications than generalized obesity — being more closely linked to insulin resistance, systemic inflammation, and accelerated atherosclerosis — and may be driven, at least in part, by glucocorticoid-induced fat redistribution. This finding challenges the exclusive use of BMI as a risk marker in this population and supports the routine incorporation of waist circumference measurement into clinical assessment.

The strong association of MetS with male sex (OR >11) is an intriguing and clinically important finding. Paradoxically, patients with MetS in this cohort were younger than those without, an observation that may reflect referral bias toward more severely affected patients at tertiary centers, earlier and more intensive immunosuppressive exposure in younger individuals, and the rising prevalence of MetS among younger men documented in contemporary epidemiological data.^7^ Collectively, these findings argue for systematic cardiometabolic surveillance across all age groups in AAV, rather than reserving screening for older patients.

The link between AAV and MetS is multifactorial. Chronic systemic inflammation promotes endothelial dysfunction, oxidative stress, and dyslipidemia. In parallel, glucocorticoid therapy — still a cornerstone of AAV management — contributes to weight gain, insulin resistance, hypertension, and adverse lipid profiles. Although the study did not identify current prednisone use as an independent predictor of MetS, the absence of cumulative exposure data precludes definitive conclusions. Notably, the higher frequency of mycophenolate mofetil use among patients with MetS was appropriately attributed to confounding by indication rather than to any direct diabetogenic or proatherogenic effect of the drug — an important methodological distinction for future studies addressing treatment patterns and metabolic consequences.

The study also underscores that AAV itself — alongside a positive family history of cardiovascular disease — constitutes an independent MetS-associated factor, reinforcing that vasculitis patients do not merely accumulate traditional risk factors at higher rates, but that the disease-specific milieu substantially amplifies the overall cardiometabolic profile. Of note, MetS prevalence did not differ by disease activity or vasculitis subtype, suggesting that cardiometabolic risk is relatively independent of the vasculitis phenotype and should be addressed in all patients regardless of disease characteristics.

The study has important limitations that the authors acknowledge transparently. Its cross-sectional design precludes causal inference, and the single-center convenience sample limits generalizability. The relatively small sample size constrains multivariable adjustment for relevant confounders, particularly cumulative glucocorticoid exposure. Prospective, multicenter, longitudinal studies are needed to clarify the temporal dynamics between AAV activity, treatment, and MetS development, and to evaluate whether MetS-directed interventions translate into reduced cardiovascular event rates in this population.^8-13^

The clinical implications are clear. Systematic screening for MetS components — blood pressure, fasting glucose, lipid profile, and waist circumference — should become standard practice in monitoring patients with AAV. Early and aggressive management of modifiable risk factors, including lifestyle interventions and glucocorticoid-sparing strategies when feasible, must be integrated into routine care. Cardiologists and rheumatologists must collaborate closely to reduce the long-term cardiovascular burden in these patients.

n conclusion, Bezerra et al.^1^ reinforce that AAV is not merely a vasculitic disease but also a cardiometabolic disease. The high prevalence of MetS — clustering with male sex, central obesity, hypertension, dysglycemia, and dyslipidemia — demands a holistic management approach that extends well beyond controlling disease activity. This study is a timely reminder that excellence in vasculitis care requires bridging the gap between immunological remission and comprehensive cardiovascular risk reduction.
